# Potency of an HIV-SAM™ vaccine in a heterologous prime-boost vaccination regimen

**DOI:** 10.1186/1742-4690-9-S2-P312

**Published:** 2012-09-13

**Authors:** K Banerjee, Y Cu, Y Sun, K Hartog, A Dey, L Brito, A Verma, A Nandi, P Sarkar, NM Valiante, AJ Geall, SW Barnett, GR Otten

**Affiliations:** 1 Novartis Vaccines and Diagnostics, Cambridge, MA, USA

## Background

Recombinant alphavirus replicon particles (VRP), carrying self-amplifying RNA, protects rhesus macaques against SHIVSF162P4 challenge when used in prime-boost regimen.

## Methods

Novartis has developed a synthetic self-amplifying mRNA (SAM™) vaccine platform that avoids limitations of cell culture production and employs synthetic non-viral vaccine delivery systems.

## Results

We evaluated systemic and mucosal immune responses in mice and rabbits using the SAM™ platform expressing HIV-1 gp140 (HIV-SAM™ vaccine) prime, protein/MF59 vaccine boost regimen for both HIV-1 Clade B and C Env antigens. In mice, the primed Env-specific IgG response to 1 µg of the HIV-SAM™ vaccine was comparable to a 10 µg dose of an identically formulated DNA vaccine, 10(7) IU of VRP, and 10 µg protein/MF59 vaccines. The HIV-SAM™ vaccine primed response could be boosted robustly by a protein/MF59 vaccine and resulted in a balanced IgG1, IgG2a subclass response, similar to that seen with the VRP vaccine, but unlike the dominant IgG1 response to protein/MF59 only vaccinations. Both Env-specific CD4+ and CD8+ T-cell responses were detectable after two HIV-SAM™ vaccinations. A TH1 type (IFNγ+, IL-5-) profile was demonstrable for the HIV-SAM™ vaccine primed, protein boosted CD4+ T-cell response, similar to that seen with the DNA or VRP primed protein boosted responses, in contrast to a TH2 type (IFNγlow, IL-5+) response seen with protein/MF59 vaccination. In rabbits, priming with the 25 or 50 µg of the formulated HIV-SAM™ vaccine induced robust and avid Env-binding IgG and HIV neutralizing antibodies that were superior to 500 µg of an unformulated DNA vaccine and comparable to VRP and protein/MF59 vaccines. In addition, protein/MF59 boostable Env-specific vaginal wash Ig was consistently demonstrable in both mice and rabbits immunized with the HIV-SAM™.

## Conclusion

Together, these results suggest that HIV-SAM™ vaccine is potent and versatile and offers potential as a novel immune priming strategy. NIAID-NIH Grant 5P01AI066287.

